# Sensitivity and specificity of speckle tracking echocardiography for coronary artery disease: a systematic review and meta-analysis

**DOI:** 10.3389/fcvm.2026.1895229

**Published:** 2026-08-28

**Authors:** Linping Xu, Haoqi Xu, Zhi Pan, Ruizhong Ye

**Affiliations:** 1 Department of Ultrasound, XianJu People's Hospital, Zhejiang Southeast Campus of Zhejiang Provincial People's Hospital, Affiliated Xianju's Hospital, Hangzhou Medical College, Taizhou, Zhejiang, China; 2 Grade 22, Chang Sha Medical University Class 2, Undergraduate Medical Imaging, Changsha, Hunan, China; 3 Emergency and Critical Care Center, Zhejiang Provincial People's Hospital, Affiliated People's Hospital, Hangzhou Medical College, Hangzhou, Zhejiang, China; 4 Department of Ultrasound Medicine, Zhejiang Provincial People's Hospital, Affiliated People's Hospital, Hangzhou Medical College, Hangzhou, Zhejiang, China

**Keywords:** coronary artery disease, meta-analysis, myocardial strain, parameters, speckle-tracking echocardiography

## Abstract

**Objectives:**

To determine the diagnostic performance of various myocardial strain parameters obtained by speckle-tracking echocardiography for detecting coronary artery disease.

**Methods:**

PubMed, Embase, Web of Science, and Cochrane Library were systematically searched from inception to 1 September 2025. Eligible studies included patients with suspected CAD, used CAG as the reference standard, and reported STE-derived strain parameters (e.g., GLS, GCS, GRS, GAS). Methodological quality was assessed via QUADAS-2, and meta-analyses were performed using a bivariate hierarchical random-effects model in STATA 18.0.

**Results:**

A total of 40 non-randomized studies involving 5,296 participants were included. The overall pooled sensitivity and specificity of STE for CAD were 77.3% (95% CI: 73.4–80.8%) and 75.0% (95% CI: 71.5–78.1%), respectively, with substantial heterogeneity across studies. Subgroup analyses suggested that global longitudinal strain (GLS) had more optimal performance (sensitivity: 78.6%, 95% CI: 74.5–82.2%; specificity: 76.3%, 95% CI: 72.7–79.7%), while global area strain (GAS) showed the highest sensitivity (83.3%, 95% CI: 74.5–89.5%) but moderate specificity (72.2%, 95% CI: 58.9–82.5%). Exercise stress STE demonstrated higher sensitivity than dobutamine stress or no stress protocols, and recovery-phase measurements showed promising but imprecise results. Deek's funnel plot indicated no significant publication bias (*P* = 0.202). GRADE assessment rated the overall certainty of evidence as low due to high risk of bias, indirectness, and imprecision.

**Conclusion:**

STE exhibits moderate diagnostic accuracy for CAD, with GLS being the most robust parameter. Future research should focus on standardizing methodologies, validating strain thresholds, and integrating STE into multidisciplinary diagnostic pathways.

**Systematic Review Registration:**

https://www.crd.york.ac.uk/PROSPERO/view/CRD420251207466.

## Introduction

Coronary artery disease (CAD) refers to a type of heart disease caused by reduced blood flow in the coronary arteries, and remains a leading cause of morbidity and mortality worldwide, with approximately 17.8 million affected individuals annually (1–3). The global burden of CAD continues to escalate, with mortality rates increasing by 22.3% from 2007 to 2017, highlighting the critical need for accurate and early diagnostic approaches (4). The current clinical standard for definitive CAD diagnosis is coronary angiography (CAG), an invasive procedure that carries inherent risks, involves radiation exposure, and incurs substantial healthcare costs (5). These limitations have driven the search for reliable, non-invasive alternatives that can accurately detect CAD.

Conventional non-invasive imaging modalities, especially conventional echocardiograph**y**, have limitations in detecting early myocardial dysfunction caused by coronary ischemia. These techniques primarily rely on visual assessment of regional wall motion abnormalities (RWMA), which is subjective and demonstrates limited sensitivity, particularly for subclinical myocardial ischemia (6). Visual assessment of RWMA can be challenging and inaccurate, creating a diagnostic gap that necessitates more objective and quantitative approaches to myocardial function assessment (7).

Speckle tracking echocardiography (STE), represents a significant advancement in cardiac ultrasound technology. The technique utilizes naturally occurring acoustic markers (speckles) within the myocardium that can be tracked frame-by-frame throughout the cardiac cycle (8). This tracking enables quantitative assessment of myocardial deformation through parameters called strain and strain rate. STE provides comprehensive evaluation of myocardial mechanics in multiple dimensions, including longitudinal strain (LS, deformation along the myocardial long axis), circumferential strain (CS, deformation in the circumferential direction), radial strain (RS, deformation across the wall thickness), and area strain (AS, derived from the combined circumferential and longitudinal strain) with the advent of three-dimensional STE (3D-STE) (9).

The clinical application of STE has gained momentum due to its ability to detect subtle myocardial dysfunction before the appearance of visible wall motion abnormalities or reduction in left ventricular ejection fraction (LVEF) (10). Global longitudinal strain (GLS) measures the percentage change in the length of myocardial fibers during the contraction phase of the heart compared to the relaxation (resting) phase. GLS has emerged as a particularly sensitive parameter for early myocardial impairment, which aligns with the anatomical vulnerability of subendocardial longitudinally-oriented fibers to ischemia (11). The 2018 American Society of Echocardiography guidelines have consequently recommended GLS as part of standard echocardiographic examination when feasible (12).

Despite numerous individual studies reporting on the diagnostic accuracy of various myocardial strain parameters obtained by STE for CAD, considerable heterogeneity exists in reported sensitivity and specificity values. For instance, recent studies have reported sensitivities ranging from 61% to 88% and specificities from 55% to 91% for various strain parameters (13). This variability may stem from differences in STE methodologies, patient populations, reference standards, and diagnostic thresholds. A systematic synthesis of the available evidence is therefore necessary to establish robust, pooled estimates of the diagnostic accuracy of STE for CAD. The aim of this study was to compare the diagnostic performance of different STE parameters: GLS and other strain for CAD detection.

## Methods

This review was conducted according to the Cochrane Handbook for Systematic Reviews of Diagnostic Test Accuracy (14), and results reported according to the preferred reporting items for systematic review and meta-analysis of diagnostic test accuracy studies (PRISMA-DTA) (15). The protocol for this review was registered in PROSPERO (CRD420251207466).

### Eligibility criteria

Two authors reviewed and chose the studies on the basis of the following inclusion criteria: (1) patients with clinical suspicion of CAD; (2) the gold standard for diagnosis of CAD is coronary angiography; (3) the diagnostic indicator is the arbitrary myocardial strain calculated by speckle tracking, e.g., GLS, global area strain (GAS), global circumferential strain (GCS), global radial strain (GRS), Territorial longitudinal strain (TLS), Territorial circumferential strain (TCS), Territorial radial strain (TRS); (4) author reported available data, e.g., No. of participants, No. of those diagnosed with CAD or significant CAD, sensitivity and specificity of myocardial strain. We excluded non-original articles, including abstracts, case reports, review articles, editorials, and expert opinions. Table 1 presents the full names and definitions or explanations of the numerous abbreviations frequently used in the article.

**Table 1:** Characteristic of included studies.

StudyID	Country	Design	Sample size	CAD patients	Age	Sex	Type of Echocardiography	Type of strain	Type of stress	Cutoff^a^	Sensitivity	Specificity	AUC (95%CI)
Akiash et al. (21)	Iran	NR	89	51	57.3 (34–88)	56/33	2DSTE	GLS	None	−19.25	0.765	0.766	0.786
Anderson et al. (22)	USA	retrospective study	146	36	59.5 (8)	104/42	2DSTE	GLS	None	−18.8	0.53	0.82	0.72
Bajracharya et al. (23)	Nepal	retrospective study	84	44	50.1 (9.5)	51/33	2DSTE	GLS	None	−18.05	0.818	0.85	0.86 (0.78, 0.95)
Biering-Sørensen et al. (24)	Denmark	NR	293	107	63.7 (9.9), 59.2 (9.7)	146/147	2DSTE	GLS	None	−18.4	0.74	0.58	0.68 (0.62, 0.74)
Biswas et al. (25)	Bangladesh	retrospective study	60	36	49 (7)	NR	3DSTE	GLS, GCS, GAS, GRS,	None	GLS >−13.5	88.9	70.8	0.840 (0.735, 0.945)
Bu et al. (26)	China	NR	271	92	57.04 (9.65)	158/113	2DSTE	GCS	None	6.35	0.76	0.91	NR
Caspar et al. (27)	France	NR	58	33	58.4 (12.8)	35/23	2DSTE	GLS	None	−19.7	0.81	0.88	0.92
Choi et al. (28)	Korea	NR	96	66	59 (9)	68/28	2DSTE	GLS	None	−19.4	0.763	0.741	0.803 (0.715, 0.890)
Dahlslett et al. (29)	Norway	NR	64	29	56 (12), 54 (12)	44/20	2DSTE	GLS	None	−21	0.93	0.78	0.90 (0.74,0.99)
Eek et al. (30)	Norway	NR	150	33	58.1 (8), 57.7 (9)	NR	2DSTE	GLS, GCS	None	GLS >−16.3	0.67	0.71	0.76 (0.67,0.85)
Elamragy et al. (31)	Egypt	prospective study	101	49	53 (8)	44/57	2DSTE	GLS	Dobutamine	−20.5	0.898	0.846	0.88 (0.82,0.94)
Hubbard et al. (33)	USA	NR	105	70	70 (8), 35 (13)	NR	2DSTE	GLS, GCS	None	GLS >−15.87	0.71	0.74	0.74
Hwang et al. (34)	Korea	NR	44	18	64 (11),66 (8)	23/21	2DSTE	GLS	Dobutamine	−19	0.706	0.833	0.81 (0.65,0.96)
Ka et al. (35)	Senegal	prospective study	52	27	62.5 (11.9)	33/19	2DSTE	GLS	None	−16.9	0.74	0.76	0.83 (0.73,0.94)
Koulaouzidis et al. (36)	Switzerland	prospective study	103	47	63.7 (9.2)	81/22	2DSTE	GLS	None	−16.3	0.66	0.732	0.692
Liu et al. (37)	China	NR	113	63	56.63 (10.21), 56.12 (8.98)	NR	2DSTE	GLS	None	−22.69	0.86	0.88	0.905 (0.810,0.947)
Mahjoob et al. (38)	Iran	NR	216	153	61.1 (9.9)	132/84	2DSTE	GLS	None	NR	0.911	0.63	0.91 (0.78,0.97)
Mansour et al. (39)	Lebanon	NR	103	12	52 (12)	84/19	2DSTE	GLS	Exercise	NR	1	0.72	NR
Marques-Alves et al. (40)	Portugal	NR	48	24	61 (12)	32/16	2DSTE	GLS	None	−17.5	0.87	0.82	0.86 (0.72,0.94)
Masada et al. (41)	Japan	NR	43	26	69 (11)	30 (13)	2DSTE	GLS	None	NR	0.762	0.5	0.6 (0.43,0.78)
Mokadem et al. (42)	Egypt	retrospective study	103	32	57.79 (8.08)	62	2DSTE	GLS	Dobutamine	−18.9	0.7812	0.8873	0.83
Montgomery et al. (43)	USA	retrospective study	123	56	60 (12)	73/50	2DSTE	GLS	Dobutamine	−17.6	0.65	0.65	0.68 (0.57,0.78)
Nucifora et al. (44)	Netherlands	NR	182	119	54 (10)	108/74	2DSTE	GLS	None	−17.4	0.83	0.77	0.83 (0.77,0.88)
Qin et al. (45)	China	NR	169	121	61.1 (11.6)	120/49	2DSTE	TLS	None	−18.7	0.59	0.69	0.70 (0.66,0.75)
Radwan et al. (46)	Egypt	NR	80	58	51.62 (8.37), 55.54 (6.45)	52/28	2DSTE	GLS	None	−15.6	0.931	0.818	0.88 (0.78,0.96)
Ragab et al. (47)	Egypt	retrospective study	125	63	58.9 (9.3), 58.3 (6.9)	86/39	2DSTE	GLS	Exercise	−18.6	0.792	0.8	0.579
Rumbinaitė et al. (48)	Lithuania	NR	81	40	64 (8.6)	45/36	2DSTE	GLS	Dobutamine	NR	0.894	0.647	0.811
Rumbinaite et al. (13)	Lithuania	NR	145	75	64.9 (7.6), 62.6 (8.8)	68/77	2DSTE	GLS, GRS, GCS, GAS, TLS, TRS, TCS	Dobutamine	NR	0.88	0.91	0.902
Sabatino et al. (49)	Italy	NR	80	50	67 (9),61 (8)	47/33	2DSTE	GLS	None	−17	0.452	0.619	NR
Saleh et al. (50)	Germany	prospective study	108	73	63 (9), 65 (12)	54/54	2DSTE	GLS, TLS	Dobutamine	GLS >−19.1	0.741	0.767	0.754
Setouhi et al. (51)	Egypt	prospective study	90	60	56.2 (13.83), 56.4 (12.16), 52.7 (12.56)	58/32	3DSTE	GLS, GAS, GCS	None	GAS>−21	0.9667	0.85	0.980 (0.925, 0.998)
Smedsrud et al. (52)	Norway	NR	86	43	64 (8), 60 (10)	43/43	2DSTE	GLS	None	−17.4	0.51	0.81	NR
Tsai et al. (53)	China	NR	152	75	63 (12)	77/75	2DSTE	GLS	None	−19	0.747	0.805	NR
Vrettos et al. (54)	UK	NR	71	61	62 (11)	49/22	2DSTE	GLS	None	−13.95	0.71	0.9	0.841 (0.750, 0.950)
Yu et al. (55)	China	retrospective study	84	42	62.24 (11.19), 64.26 (10.19)	45/39	2DSTE	GLS	None	−19.7	0.7619	0.5476	0.685
Zhang et al. (56)	China	NR	139	110	52.8 (5.9), 55.4 (6.0),53.6 (5.9)	111/28	2DSTE	GLS	None	−21.35	0.72	0.84	0.846
Zhang et al. (57)	China	NR	195	165	63 (9)	109/86	2DSTE	GLS	None	−17.3	0.38	0.97	0.69
Zhu et al. (58)	China	NR	690	346	64.19 (9.88), 64.22 (9.22)	386/304	2DSTE	GLS	None	−17.95	0.737	0.63	0.727 (0.688, 0.767)
Zuo et al. (59)	China	NR	143	88	56 (7)	65/78	2DSTE	GLS	None	−17.75	0.725	0.608	0.769
Zuo et al. (60)	China	NR	211	145	60.52 (9.59), 57.00 (8.40),53.02 (10.06)	132/79	2DSTE	GLS	None	−19.05	0.781	0.727	0.875 (0.819, 0.932)

a When the study involves multiple strain types, here we report the cutoff value, sensitivity, specificity and AUC (95% CI) of the strain parameter with the largest AUC.

### Search strategy and study selection

PubMed, Embase, Web of Science, Cochrane Library were searched from inception to 1 September 2025. The full search strategy attached in e-Appendix 1 (Supplementary Table 1). The standard screening process consists of two steps. The first step involves reading the questions and abstracts to eliminate obviously irrelevant literature. The second step involves reading the full texts to determine whether the studies should be included or excluded. All of the above were independently completed by the two authors using EndNote X9. Any differences were resolved through discussion.

### Data extraction and assessment of methodological quality

Data extraction was independently done by two authors and disagreements discussed with a third author. The following information were extracted from the included studies: last name of first author, publication year, study design, sample size, No. of patients with CAD, age of participants, gender proportion of participants, type of Echocardiography, type of stress, type of myocardial strain, cutoff of myocardial strain, sensitivity of myocardial strain, specificity of myocardial strain and area under receive operator characteristic (ROC) curve of myocardial strain. If the data cannot be obtained, the corresponding author were contacted via email to request them to provide supplementary data. Additionally, two reviewers assessed the risk of bias of included studies using the QUADAS-2 tool.

### Data synthesis and statistical analyses

The meta-analysis was performed employing a bivariate random-effects modeling framework (16). This approach generated a combined sensitivity and specificity estimate, presented graphically as a summary point with corresponding 95% confidence and prediction regions on a summary receiver operating characteristic (SROC) curve. Pre-specified subgroup analyses evaluated variations in diagnostic performance across categories including type of stress, type of strain study design, and type of measuring status, ROB of study, cutoff of myocardial strain, diagnostic thresholds, diagnostic technique. For each subgroup, pooled sensitivity and specificity were computed, SROC curves with summary points and confidence intervals were generated (17), and between-subgroup differences were tested for statistical significance using likelihood ratio tests. All meta-analyses were executed in STATA 18.0 (College Station, TX: StataCorp LLC) utilizing the “metadta” package (18). Publication bias and small-study effects were assessed by constructing Deek's funnel plot and performing the corresponding statistical test for asymmetry via the *midas* command (19). To evaluate the robustness of the findings, sensitivity analyses were performed by excluding studies at high risk of bias. Finally, the certainty of the evidence for the main findings was appraised using the GRADE methodology, with results visualized in GRADEpro GDT (20).

## Results

**Figure 1:**
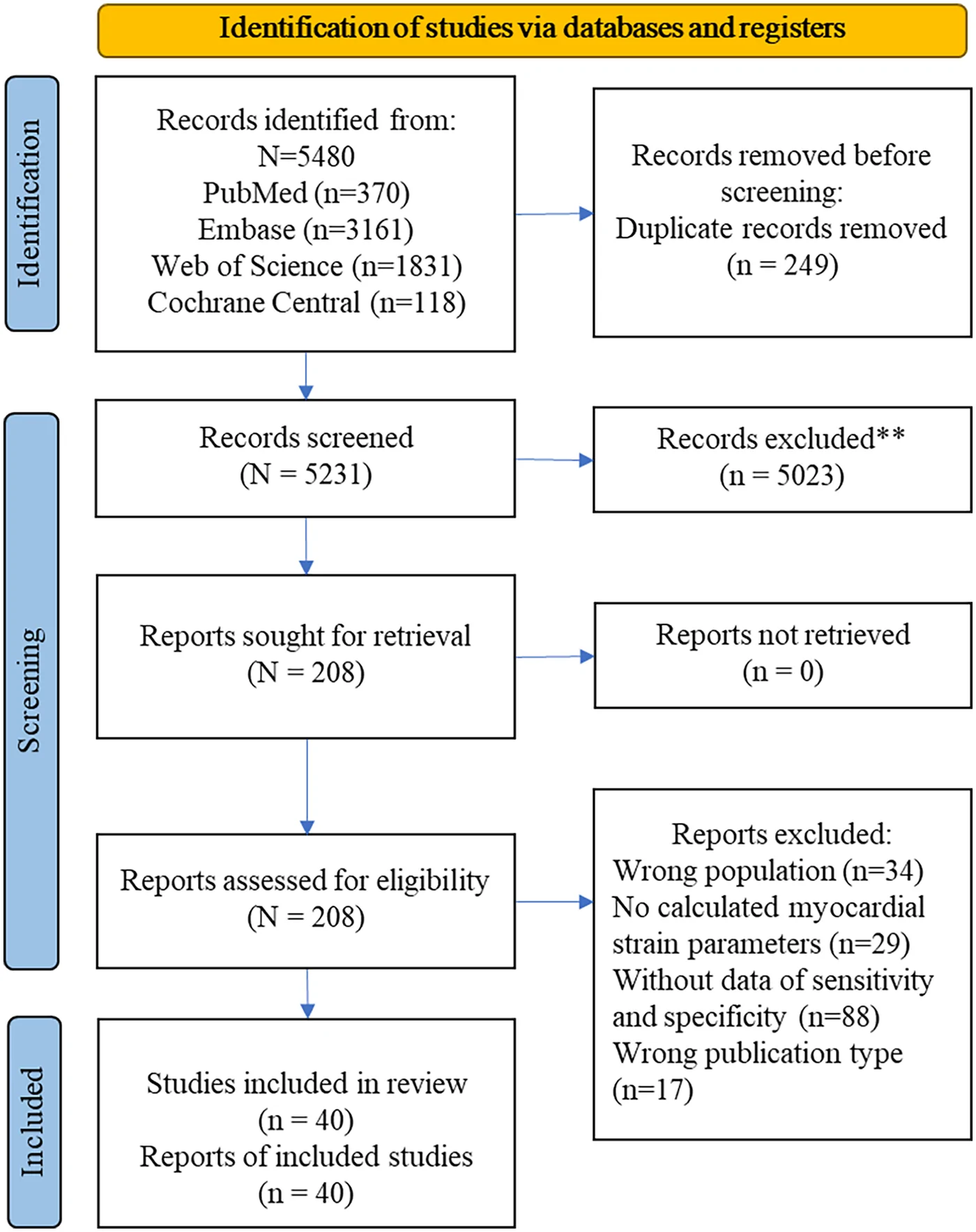
Flowchart of selection process.

A total of 5,480 records were retrieved from four databases, namely PubMed, Embase, Web of Science, Cochrane Central. We removed 249 duplicated records initially. After title and abstract screening and full-text screening, 40 studies (13, 21–60) were included in the meta-analysis. Figure 1 showed the detailed selection process.

### Characteristics of included studies

A total of 40 nonrandomized studies with 5 296 participants were included in the present systematic review and meta-analysis. Publication year of the 40 included studies ranged from 2009 to 2025. The 40 studies were conducted in 21 countries. The countries with more than one study included China, Egypt, the United States, Norway, Iran, South Korea, and Lithuania. Average age of participants in these studies ranges from 35 to 70. The positive rate of CAD is from 11.65% to 90.45%. Two studies employed 3DSTE to analyze cardiac strain, while the rest of 38 studies employed 2DSTE. Seven studies conducted dobutamine stress echocardiography, two studies conducted exercise stress echocardiography, and the rest of 31 included studies did not perform echocardiography with stress. Thirty-eight studies calculated GLS, 6 studies calculated GCS, 5 studies calculated TLS, less than 5 studies calculated GAS, GRS, TCS, TRS. Detailed information of included studies is showed in Table 1.

### Quality assessment of included studies

**Figure 2:**
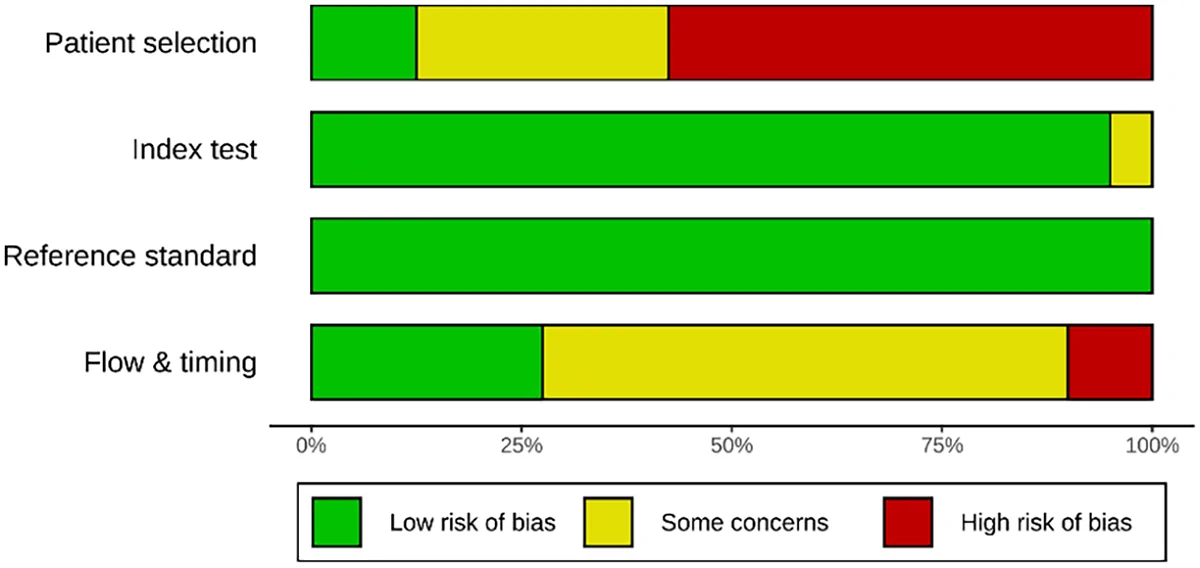
Summary graph of risk of bias.

According to the QUADAS-2 tool, a total of 24 studies (60.0%) had high risk of bias, 11 studies (27.5%) had some concerns, and 5 studies (12.5%) had low risk of bias. In terms of patient selection, due to non-random sampling, 24 studies (60.0%) have a high risk of bias. In the index test aspect, only 2 studies (5.0%) have some concerns, and there is no high bias risk. In the reference standard aspect, since we only included studies using coronary angiography as the gold standard, there is no high bias risk or some concerns. In the flow and timing aspect, 4 studies (10.0%) have a high bias risk, 25 studies (62.5%) have some concerns, and 11 studies (27.5%) have a low risk of bias (Figure 2).

### Summary of findings

**Figure 3:**
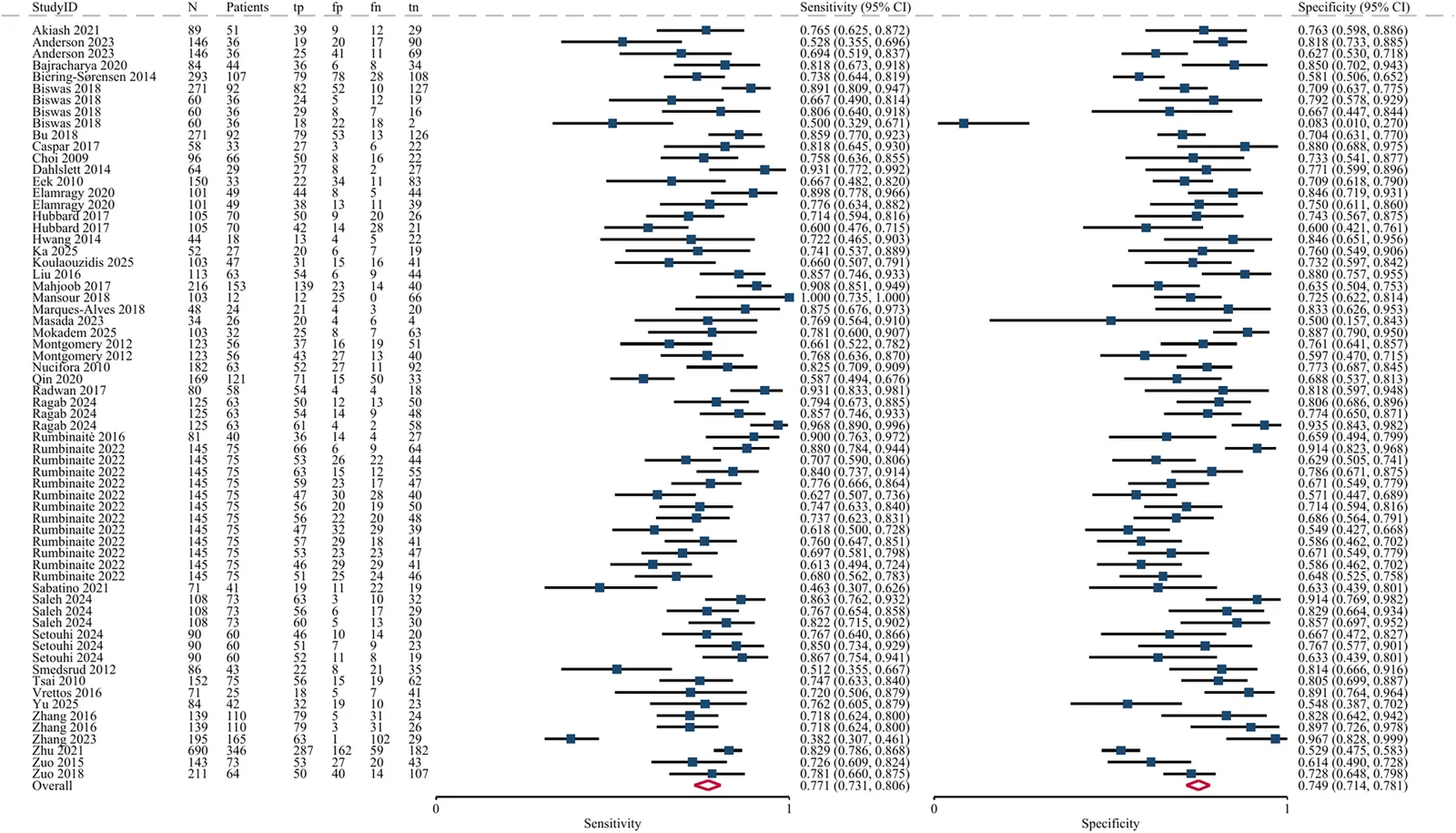
Forest plot of sensitivity and specificity of all included studies.

**Figure 4:**
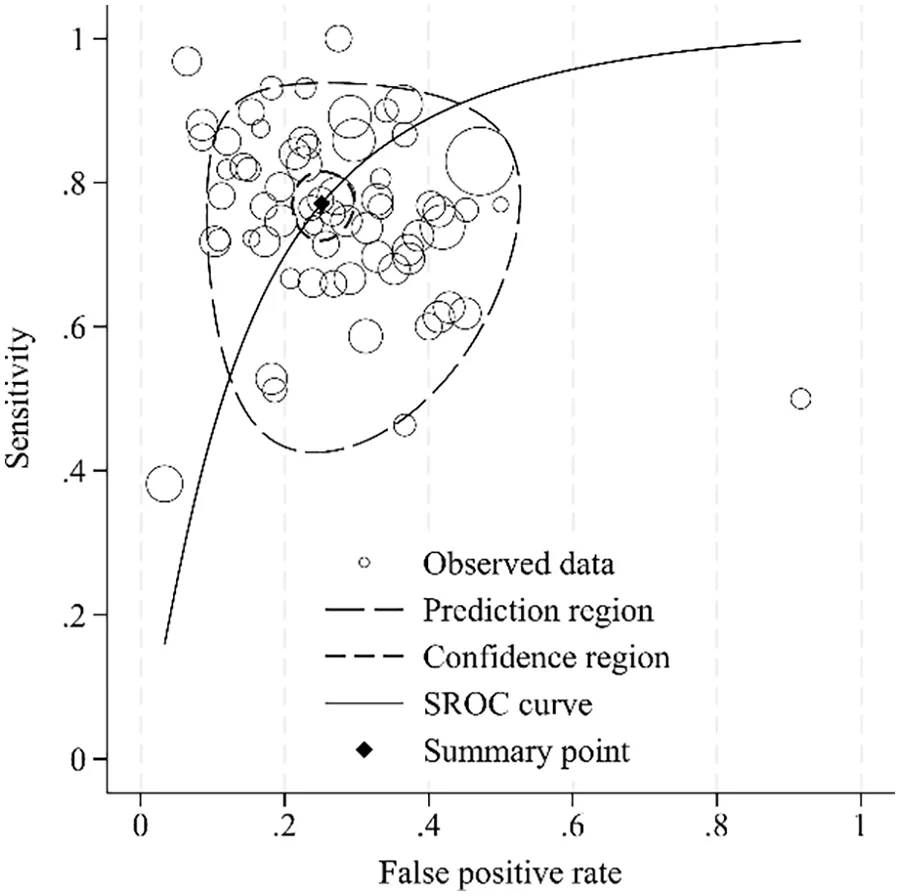
SROC plot of sensitivity and specificity of all included studies.

**Figure 5:**
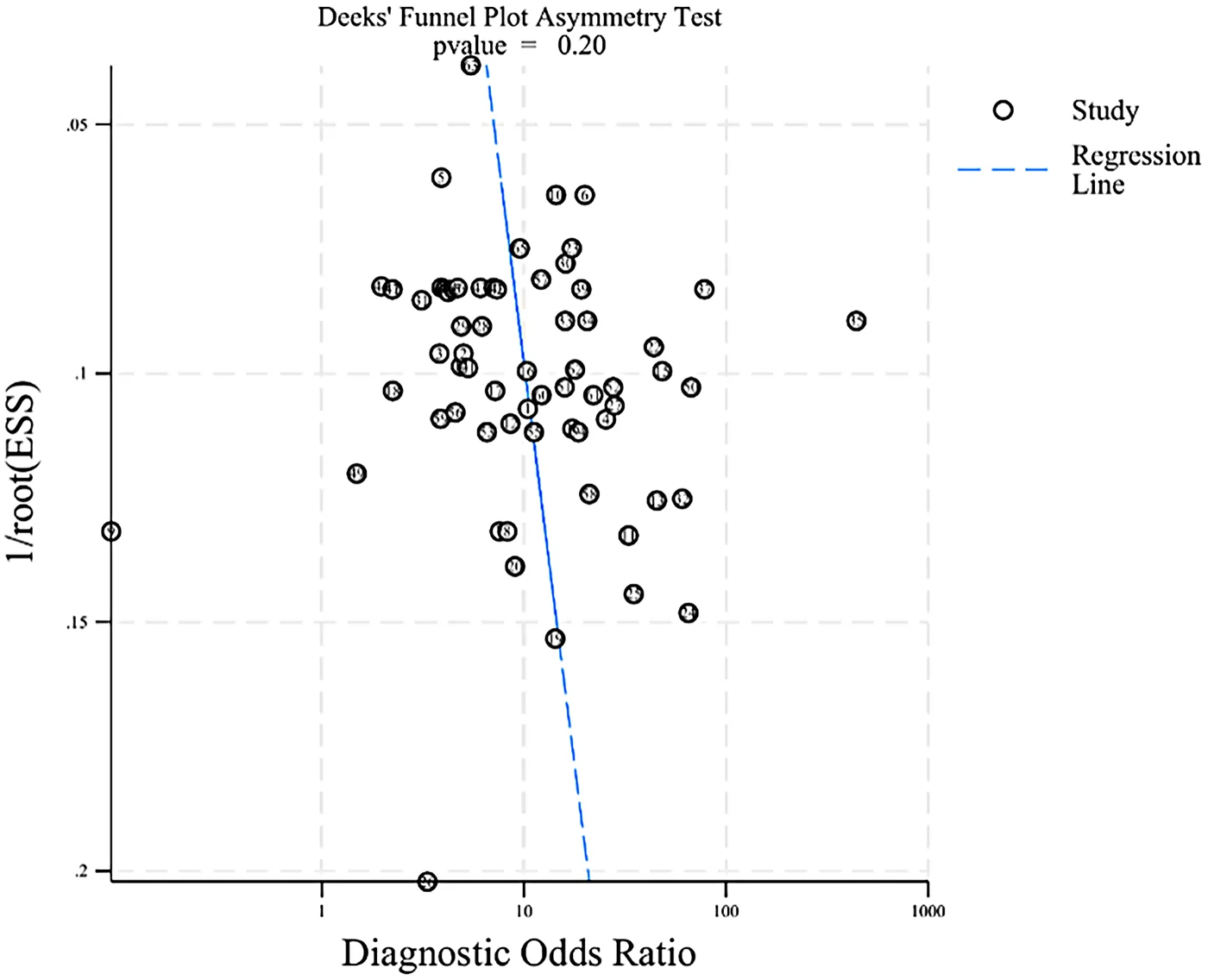
Deeks' funnel plot of asymmetry test. ESS Effective Sample Size.

Figure 3 showed that in 40 studies, sensitivities ranged from 38% to 100% and specificities ranged from 8% to 97%. The results are presented in chronological order to assess the possible evolving trends that may arise as a result of technological advancements or changes in diagnostic criteria; however, based on the publication years, no significant trend changes were observed overall. The summary sensitivity was 77.3% (95%CI 73.4%–80.8%) and the summary specificity was 75.0% (95%CI 71.5%–78.1%). Figure 4 illustrated a wide 95% prediction area in the SROC plot. Table 2 presents the main findings of this study and the GRADE assessment. The meta-analysis had a low certainty due to Deek's funnel plot was employed to assess whether there were publication bias and other factors related to sample size. The Deeks' funnel plot displayed diagnostic odds ratio against the inverse square root of the effective sample size (1/√ESS). The funnel plot showed good symmetry, with a *p*-value of 0.202, indicating the absence of publication bias or the influence of small sample size studies (Figure 5).

**Table 2:** Summary of findings.

Review question: For patients suspected of having CAD, how accurate are the myocardial strain parameters obtained through speckle-tracking in diagnosing CAD?				
Population: participants with suspected CAD Setting: hospitals Study design: Prospective studies, retrospective studies Index test: myocardial strain obtained by speckle-tracking (GLS, GAS, GRS, GCS, TLS, TRS, TCS) Target Condition: CAD eference Standards: coronary angiography Limitations in the evidence High risk of bias, mainly driven by patient selection Patient selection: 24 studies (60.0%) had high risk of bias or some concerns Index test: two with some concerns, no high risk of bias Reference Standard: no high risk of bias or some concerns Flow and timing: 4 studies (10.0%) have a high bias risk, 25 studies (62.5%) have some concerns Overall assessment: 24 studies (60.0%) were judged to be at high risk of bias				
Findings				
No. of studies (Participants)	Median proportion with target condition % (IQR)	Overall sensitivity % (95%CI	Overall specificity % (95%CI)	GRADE Certainty of Evidence
40 (5,296)	52.15 (47.19, 66.67)	77.3% (73.4 to 80.8%)	75.0% (71.5 to 78.1%)	⊕⊕◯◯ Low

### Subgroup analyses

We conducted subgroup analyses to identify sources of heterogeneity and to assess the effect of different study characteristics on the diagnostic performance of CAD in patients with suspected CAD. Table 3 describes subgroup analyses for key study characteristics. Forest plots of all subgroup analyses are showed in Supplementary Figure S1-S8.

**Table 3:** Subgroup analysis of diagnostic performance of different study characteristic.

Subgroup	No. of study	No. of participants	Sensitivity (95%CI)	*P* value	Specificity (95%CI)	*P* value
Study Design				0.200		0.142
Prospective	5	936	0.806 (0.770, 0.838)		0.777 (0.707, 0.834)	
Retrospective	7	454	0.774 (0.705, 0.831)		0.753 (0.665, 0.824)	
Type of strain				0.005		<0.001
GLS	38	5,000	0.786 (0.745, 0.822)		0.763 (0.727, 0.797)	
GCS	6	821	0.740 (0.643, 0.817)		0.667 (0.618, 0.712)	
GRS	2	205	0.737 (0.669, 0.795)		0.652 (0.576, 0.721)	
GAS	2	150	0.833 (0.745, 0.895)		0.722 (0.589, 0.825)	
TLS	3	422	0.708 (0.602, 0.795)		0.727 (0.618, 0.814)	
TCS	1	145	0.616 (0.536, 0.690)		0.567 (0.485, 0.647)	
TRS	1	145	0.720 (0.643, 0.786)		0.617 (0.534, 0.693)	
Type of stress				0.016		0.678
None	35	5,057	0.757 (0.713, 0.796)		0.743 (0.705, 0.778)	
Dobutamine	8	698	0.803 (0.760, 0.839)		0.784 (0.703, 0.847)	
Exercise	2	128	0.880 (0.785, 0.936)		0.745 (0.670, 0.808)	
Type of measuring status				0.723		0.041
Peak	39	5,445	0.776 (0.736, 0.812)		0.744 (0.707, 0.777)	
Rest	4	480	0.732 (0.629, 0.814)		0.800 (0.744, 0.847)	
Recovery	3	314	0.856 (0.641, 0.952)		0.824 (0.600, 0.936)	
Rob				0.060		0.102
High risk	41	5,349	0.746 (0.701, 0.792)		0.717 (0.672, 0.763)	
Some concerns	14	2,224	0.762 (0.707, 0.817)		0.729 (0.655, 0.802)	
Low risk	10	910	0.816 (0.776, 0.857)		0.792 (0.739, 0.845)	
Cutoff of myocardial strain				0.212		
< −18	19	2,318	0.782 (0.741, 0.822)		0.782 (0.728, 0.835)	
>= −18	15	2,570	0.721 (0.633, 0.808)		0.737 (0.671, 0.804)	
Diagnostic Thresholds				0.400		0.194
50%	25	3,589	0.736 (0.680, 0.791)		0.728 (0.674, 0.782)	
70%	11	1,383	0.800 (0.740, 0.859)		0.800 (0.722, 0.877)	
70% or LM>=50%	24	3,050	0.763 (0.710, 0.815)		0.687 (0.621, 0.754)	
75%	2	233	0.823 (0.673, 0.973)		0.745 (0.604, 0.886)	
Technique				0.578		0.203
2DSTE	65	7,803	0.757 (0.724, 0.791)		0.743 (0.709, 0.776)	
3DSTE	7	721	0.783 (0.700, 0.866)		0.614 (0.419, 0.809)	

Five prospective studies with 936 participants derived a favorable sensitivity (0.806, 95%CI: 0.770–0.838) and a decent summary specificity (0.777, 95%CI: 0.707–0.834), while seven retrospective studies with 454 participants had a lower sensitivity (0.774, 95%CI: 0.705–0.831) and a lower specificity (0.753, 95%CI: 0.665–0.824). However, difference of sensitivity (*P* = 0.200) or specificity (*P* = 0.142) was not statistically significant between-group.

Among the seven myocardial strain types, the ranking of the sensitivity of summary is GAS, GLS, GCS, GRS, TRS, TLS, TCS. The ranking of the specificity of summary is GLS, TLS, GAS, GCS, GRS, TRS, TCS. Although there were significant differences in sensitivity(*P* = 0.005) or specificity (*P* < 0.001), in general, the diagnostic performance of global myocardial strain is superior to that of local myocardial strain. Among them, GLS has good sensitivity (0.786, 95%CI: 0.745–0.822) and specificity (0.763, 95%CI: 0.727–0.797). Secondly, GAS has the best sensitivity (0.833, 95%CI: 0.745–0.895) and a decent specificity (0.722, 95%CI: 0.589–0.825).

For type of stress, there was significant difference in sensitivity (*P* = 0.016). The summary sensitivity of myocardial strain under exercise stress are higher 12.3% than myocardial strain without stress, and are higher 7.7% than myocardial strain under dobutamine stress. For different type of stress, there was no significant difference in the specificity of myocardial strain. Nevertheless, for type of measuring status, there was significant difference in specificity. Results suggested that diagnostic performance of myocardial strain at recovery (sensitivity: 0.856, specificity: 0.824) was higher than at peak (sensitivity: 0.776, specificity: 0.744) and at rest (sensitivity: 0.732, specificity: 0.800), but sensitivity and specificity of myocardial strain at recovery had a wide 95%CI (sensitivity: 0.641–0.952, specificity: 0.600–0.936).

In addition, based on the risk of bias, cutoff of myocardial strain, diagnostic thresholds, and diagnostic techniques of the study, we conducted subgroup analyses. Although the results showed that studies with low bias risk had higher sensitivity and specificity when GLS was less than −18% and the degree of vascular stenosis was higher than 70%, the results of the heterogeneity test among the groups showed no statistically significant differences (*P* > 0.05).

### Sensitivity analyses

Sensitivity analyses were conducted by removing studies with high risk of bias. Risk of bias was assessed by the QUADAS-2 tool, and judged upon four domains: patient selection, index test, reference standard and flow and timing. Sensitivity analysis was conducted by removing 24 studies with high risk of bias in multiple domains. We pooled the rest of 16 studies with low risk of bias or some concerns, which includes 2,311 participants and had approximately 50.01% CAD patients. The pooled result showed that summary sensitivity was slightly increased, and summary specificity was slightly decreased (Table 4).

**Table 4:** Sensitivity analysis of removing studies with high risk of bias.

Studies or subgroup	Number of studies (Participants)	Median proportion with target condition % (IQR)	Summary sensitivity % (95% confidence interval)	Summary specificity % (95% confidence interval)
All studies	40 (5,296)	52.15 (47.19, 66.67)	77.3 (73.4 to 80.8)	75.0 (71.5 to 78.1)
After removing studies with high risk of bias	16 (2,311)	50.01 (45.61, 66.90)	78.5 (73.4 to 82.9)	74.7 (69.1 to 79.6)

## Discussion

The systematic review assessed the available evidence on myocardial strain analysis of speckle tracking echocardiography for the diagnosis of coronary artery disease. Forty relevant studies were included in the review, with 5,296 patients. The overall summary estimate for sensitivity and specificity was 77.3% (95%CI 73.4%–80.8%) and 75.0% (95%CI 71.5%–78.1%), respectively, but must be interpreted with caution given the substantial heterogeneity between studies.

### Diagnostic value of myocardial strain obtained by STE for CAD

The moderate diagnostic performance of myocardial strain obtained by STE can be attributed to its ability to classify CAD and non-CAD (non-significant CAD). Among strain parameters, GLS demonstrated the most consistent performance, with a sensitivity of 78.6% (95%CI: 74.5% to 82.2%) and specificity of 76.3% (72.7% to 79.7%). This result is superior to the findings of Liou et al. In Liou's research published in 2016, the sensitivity of GLS in diagnosing CAD was 74.4%, and the specificity was 72.1% (61). The possible reasons for these differences include: (1) In Liou's study, the resting-state parameter values were used, while in this study, GLS values for both the resting state, recovery state, and peak state were included; (2) Liou's study has been conducted for nearly 10 years. During this period, not only has 2DST technology made certain advancements, but 3DST technology has also undergone significant development, and this study has incorporated the diagnostic values of GLS calculated using 3DSTE.

This study was the first to investigate the diagnostic value of myocardial strain parameters other than GLS, such as GAS, GCS, GRS, et al. We found that GAS exhibited the highest sensitivity (83.3%), though its specificity was lower (72.2%), suggesting a role for GAS in early screening or ruling out CAD in high-risk populations. At present, no literature has provided a reasonable explanation for this. Therefore, this might be related to the small amount of research involved. Thus, this result should be treated with caution.

When discussing the sources of heterogeneity, the research design can be one of the possible sources of heterogeneity. However, since many studies did not report their research types, no significant result could be obtained. Secondly, the measuring status also has certain influences on the heterogeneity. In previous studies, GLS typically increased from rest to peak dose in patients with no CAD, while GLS usually decreased in patients with CAD (62–64). This indicates that there is a greater difference in GLS at peak between the patient group and the control group. That is to say, GLS at peak has higher sensitivity than at rest. Additionally, Liang et al.'s research indicates that the left ventricular strain index can determine the location of myocardial damage (65). Another study by Notten et al. evaluated the strain values and strain rates of 42 athletes during intense exercise, and found that the myocardial damage caused by exercise was related to the decrease in strain values (66). The above two studies indicate that stress may increase the degree of myocardial strain in patients with CAD.

In addition, although the trends align with clinical expectations—more stringent quality control, more specific strain thresholds, and more definitive anatomical stenosis criteria potentially improving diagnostic accuracy—the differences between groups were not significant, suggesting that these individual factors may not be the primary source of heterogeneity in this study. The limited number of studies included within each subgroup (particularly in the low bias-risk group) may have resulted in insufficient statistical power, preventing the detection of true differences. Furthermore, substantial heterogeneity still exists among studies regarding ultrasound equipment models, analysis software, strain measurement methods (2DSTE vs. 3DSTE), and patient population characteristics (such as concomitant medications and history of myocardial infarction), and these confounding effects may have masked the influence of individual subgroup variables on the overall results.

Nevertheless, the above subgroup analysis results provide methodological guidance for clinical practice: when feasible, preference should be given to evidence from studies with low bias risk, using GLS ≤ −18% as one threshold for suspected obstructive CAD, in conjunction with anatomical criteria such as ≥70% stenosis for a comprehensive assessment. However, due to the lack of statistical significance in these differences, we emphasize that these findings should be considered exploratory and require validation in larger prospective studies with standardized protocols. Overall, the pooled results of this study still support STE as an effective non-invasive tool for detecting coronary artery disease, but clinical decisions should integrate patient symptoms, stress testing, and functional revascularization indicators (such as FFR) for a holistic evaluation (67).

### Comparison with other non-invasive examination methods

STE has many advantages. Unlike CAG, it does not require invasive procedures, was cost-effective, and does not produce radiation (68). Compared with traditional echocardiography that relies on subjective visual assessment of RWMA, STE provides more objective and quantitative strain data and has better repeatability (69).

Emerging non-invasive technologies, such as pulse contour imaging (PCG), have also been used in the diagnostic research of CAD. A recent meta-analysis indicates that the CAD scoring system based on PCG has 87% sensitivity but only 35% specificity, suggesting that its main purpose is as an exclusion diagnostic tool (70). In contrast, STE provides balanced sensitivity and specificity, making it suitable for screening and confirmatory diagnosis.

Advanced imaging techniques, such as coronary computed tomography angiography (CCTA) and cardiac magnetic resonance (CMR), can directly describe plaque features and assess ischemia. However, they involve radiation (CCTA) or high costs (CMR), limiting their routine use. STE, especially with the emergence of 3D-STE, helps fill this gap and enables detailed myocardial mechanics assessment in the clinical setting (71).

### Combination with invasive and artificial intelligence-based methods

The limitations of STE, such as operator dependence and image quality requirements, are being addressed through technological innovation. For example, frameworks like Plaque-Cap, based on artificial intelligence, utilize visual language models to generate automated and easily understandable descriptions of atherosclerotic plaques from intravascular ultrasound (IVUS) images (72). Although IVUS is an invasive examination method, these tools highlight the potential of combining quantitative imaging with artificial intelligence to improve diagnostic accuracy and reporting efficiency.

Invasive imaging techniques like intravascular ultrasound (IVUS) and optical coherence tomography (OCT) can provide high-resolution descriptions of plaque features and the identification of characteristics such as thin-cap fibroatheroma (TCFA) and lipid arc extension. However, their invasive nature limits their widespread use in initial diagnosis. STE can be used as a screening method for invasive detection, especially in patients with low to moderate risk.

Furthermore, related research is exploring the role of strain imaging in guiding treatment. For example, the V-ACCELERATE trial uses IVUS and OCT to evaluate the impact of ezetimibe on the progression of coronary artery plaques in patients with acute myocardial infarction. Similarly, the DCB-IVUS trial studies the application of IVUS-guided drug-coated balloon angioplasty in small-vessel coronary artery disease (73). In these cases, STE can be used for non-invasive monitoring of treatment responses.

### Clinical significance and future directions

The research results of this review support the inclusion of myocardial strain (especially GLS) in the routine echocardiography examination process for coronary artery disease assessment. The 2018 American Society of Echocardiography guidelines have already recommended the use of GLS for detecting subclinical myocardial injury, and our research results further support this recommendation.

However, there are still some challenges: (1) Standardization: There is an urgent need to establish standardized thresholds for strain parameters for different vendors and populations. (2) Training: To achieve widespread adoption, training for ultrasound technicians and cardiologists in STE acquisition and interpretation is required. (3) Technological progress: 3D-STE and layer-specific strain analysis may further improve diagnostic accuracy, but they need to be validated in larger cohorts.

Future research should focus on the following aspects: 1) Multicenter prospective studies: aimed at establishing standardized strain thresholds and validating the diagnostic criteria for STE-based strain parameters in different populations. 2) Integration of artificial intelligence: using machine learning techniques to automate strain analysis and enhance reproducibility. 3) Outcome studies: evaluating the impact of management approaches based on STE on key clinical indicators such as mortality and hospitalization rates.

### Limitations

This review has some limitations. Firstly, significant heterogeneity was observed, and we attempted to address this issue through subgroup analysis, but we were unable to fully resolve it. Secondly, many of the included studies had a high risk of bias, especially in terms of patient selection. Thirdly, the sample sizes for certain strain parameters (such as GAS, TRS) and stress protocols (such as exercise) were limited, which prevented us from drawing clear conclusions for these subgroups. Finally, although no statistical significance was reached, we cannot completely rule out the possibility of publication bias.

## Conclusion

In summary, STE demonstrates moderate diagnostic accuracy for CAD, with GLS emerging as the most robust parameter. Future efforts should focus on standardizing methodologies, validating strain cutoffs, and integrating STE into multidisciplinary CAD diagnostic pathways.

## Data Availability

The original contributions presented in the study are included in the article/Supplementary Material, further inquiries can be directed to the corresponding author.
