# Supplementary material for: Sensitivity and specificity of speckle tracking echocardiography for coronary artery disease: a systematic review and meta-analysis
Source: Front Cardiovasc Med. 2026 Aug 28;13:1895229. doi: 10.3389/fcvm.2026.1895229 (PMC13561902; doi:10.3389/fcvm.2026.1895229)
Supplement: Supplementary file 1 [file Supplementaryfile1.docx]

electronic supplementary material

Tabla OF contenido

[e-Appendix 1. Search Strategy	2](#_Toc10908)

[e-Appendix 2. Subgroup Analyses	4](#_Toc6172)

[Fig. S1 Forest plot of subgroup analysis by study design.	4](#_Toc34)

[Fig. S2 SROC plot of subgroup analysis by study design.	4](#_Toc23466)

[Fig. S3 Forest plot of subgroup analysis by type of strain.	5](#_Toc24248)

[Fig. S4 SROC plot of subgroup analysis by type of strain.	5](#_Toc25426)

[Fig. S5 Forest plot of subgroup analysis by type of stress.	6](#_Toc37)

[Fig. S6 SROC plot of subgroup analysis by type of stress.	6](#_Toc28147)

[Fig. S7 Forest plot of subgroup analysis by type of measuring status.	7](#_Toc19239)

[Fig. S8 SROC plot of subgroup analysis by measuring status.	7](#_Toc9570)

# e-Appendix 1. Search Strategy

**PubMed**

| **Search** | **Query** | **Results** |
| --- | --- | --- |
| **#1** | speckle tracking[tiab] OR Velocity Vector Imaging[tiab] OR edge tracking[tiab] OR longitudinal strain[tiab] OR circumferential strain[tiab] OR radial strain[tiab] | **15,284** |
| **#2** | Coronary Artery Disease[mh] OR Coronary Artery Diseases[tiab] OR Coronary Arteriosclerosis[tiab] OR Arterioscleroses, Coronary[tiab] OR Coronary Arterioscleroses[tiab] OR Coronary Atherosclerosis[tiab] OR Coronary Atheroscleroses[tiab] OR Left Main Coronary Artery Disease[tiab] OR Left Main Coronary Disease[tiab] OR Left Main Disease[tiab] OR Left Main Diseases[tiab] | **104,753** |
| **#3** | #1 AND #2 | **370** |

Embase

| **Search** | **Query** | **Results** |
| --- | --- | --- |
| **#1** | ' speckle tracking':ti,ab,kw OR 'Velocity Vector Imaging':ti,ab,kw OR 'edge tracking':ti,ab,kw OR 'longitudinal strain':ti,ab,kw OR 'circumferential strain':ti,ab,kw OR 'radial strain':ti,ab,kw | **34909** |
| **#2** | 'Coronary Artery Disease'/exp OR 'Coronary Artery Diseases':ti,ab,kw OR 'Coronary Arteriosclerosis':ti,ab,kw OR 'Coronary Arterioscleroses':ti,ab,kw OR 'Coronary Atherosclerosis':ti,ab,kw OR 'Coronary Atheroscleroses':ti,ab,kw OR 'Left Main Coronary Artery Disease':ti,ab,kw OR 'Left Main Coronary Disease':ti,ab,kw OR 'Left Main Disease':ti,ab,kw OR 'Left Main Diseases':ti,ab,kw | **485345** |
| **#3** | #1 AND #2 | **3161** |

Web of science

| **Search** | **Query** | **Results** |
| --- | --- | --- |
| **#1** | TS=(speckle tracking OR Velocity Vector Imaging OR edge tracking OR longitudinal strain OR circumferential strain OR radial strain) |  |
| **#2** | TS=( Coronary Artery Diseases OR Coronary Arteriosclerosis OR Arterioscleroses, Coronary OR Coronary Arterioscleroses OR Coronary Atherosclerosis OR Coronary Atheroscleroses OR Left Main Coronary Artery Disease OR Left Main Coronary Disease OR Left Main Disease OR Left Main Diseases) |  |
| **#3** | #1 AND #2 | **1831** |

Cochrane Library

| **Search** | **Query** | **Results** |
| --- | --- | --- |
| #1 | (speckle tracking OR Velocity Vector Imaging OR edge tracking OR longitudinal strain OR circumferential strain OR radial strain ):ti,ab,kw | 1733 |
| #2 | MeSH descriptor: [Coronary Artery Disease] explode all trees | 9665 |
| #3 | (Coronary Artery Diseases OR Coronary Arteriosclerosis OR Arterioscleroses, Coronary OR Coronary Arterioscleroses OR Coronary Atherosclerosis OR Coronary Atheroscleroses OR Left Main Coronary Artery Disease OR Left Main Coronary Disease OR Left Main Disease OR Left Main Diseases):ti,ab,kw | 12488 |
| #4 | #2 OR #3 | 19181 |
| #5 | #1 AND #4 | 118 |

# e-Appendix 2. Subgroup Analyses

Fig. S1 Forest plot of subgroup analysis by study design.

Fig. S2 SROC plot of subgroup analysis by study design.

Fig. S3 Forest plot of subgroup analysis by type of strain.

Fig. S4 SROC plot of subgroup analysis by type of strain.

Fig. S5 Forest plot of subgroup analysis by type of stress.

Fig. S6 SROC plot of subgroup analysis by type of stress.

Fig. S7 Forest plot of subgroup analysis by type of measuring status.

Fig. S8 SROC plot of subgroup analysis by measuring status.
